# Creating a Usable and Effective Digital Intervention to Support Men to Test for HIV and Link to Care in A Resource-Constrained Setting: Iterative Design Based on A Person-Based Approach and Human Computer Interaction Methods

**DOI:** 10.2196/65185

**Published:** 2025-04-17

**Authors:** Anya Zeitlin, Thulile Mathenjwa, Thembelihle Zuma, Sally Wyke, Philippa Matthews, Nuala McGrath, Janet Seeley, Maryam Shahmanesh, Ann Blandford

**Affiliations:** 1 UCL Interaction Centre University College London London United Kingdom; 2 Africa Health Research Institute Durban South Africa; 3 School of Public Health University of Cape Town Cape Town South Africa; 4 School of Nursing and Public Health University of KwaZulu Natal Durban South Africa; 5 School of Social and Political Sciences University of Glasgow Glasgow United Kingdom; 6 Faculty of Medicine and Faculty of Social Sciences University of Southampton Southampton United Kingdom; 7 London School of Hygiene & Tropical Medicine London United Kingdom; 8 Institute for Global Health University College London London United Kingdom

**Keywords:** person-based approach, HIV, resource-constrained settings, digital intervention, user-centered design, behavior change techniques, digital health

## Abstract

**Background:**

It is challenging to design usable and effective digital health interventions (DHIs). The person-based approach (PBA) has been proposed to incorporate users’ perspectives for the design of DHIs. However, it does not explicitly describe the iterative stages of design and evaluation that are essential in moving from early planning to deployment. For this, we draw on methods from human computer interaction (HCI) that have been developed for various situations.

**Objective:**

This study aimed to reflect on the adaptation and synthesis of PBA and HCI approaches to developing DHIs. We present a case study applying both approaches to develop Empowering People through Informed Choices for HIV (EPIC-HIV1), a DHI designed for men living in rural KwaZulu-Natal, South Africa, intended to support them in making an informed choice about whether to take an HIV test and, if necessary, engage in care.

**Methods:**

We conducted a retrospective analysis of the documentation generated during the development of EPIC-HIV1 including findings about requirements, design representations, and the results of iterative rounds of testing. We developed an account of the process, the outcomes, and the strengths and limitations of the design and evaluation techniques applied. We also present the design of EPIC-HIV1 and summarize considerations when designing for hard-to-reach people in such settings.

**Results:**

The PBA was applied to deliver a first prototype. This helped identify key messages to convey and how to manage issues such as user privacy, but the resulting prototype was judged by the team not to be engaging for potential users, and it was unclear whether the design was inclusive of people with low digital or health literacy. We therefore introduced methods from HCI to iteratively test and refine the app. Working with local community representatives, we conducted four refinement cycles with 29 participants, adapting and retesting the app until no further changes were needed. Key changes included making it clearer what the consequences of selecting options in the app were and changing wording to minimize misconceptions (eg, that the app would test for HIV) while addressing common concerns about testing and emphasizing long-term benefits of engaging with care, if needed.

**Conclusions:**

Techniques for developing DHIs need to be situationally appropriate. The PBA enabled us to establish both empirical data and theory to design the content of EPIC-HIV1, but it did not directly inform interaction design to make the app usable and effective for the intended users; HCI techniques tailored to the setting enabled us to refine the app to be easy for men with little familiarity with digital technologies to use within the constraints of the setting. Iterative testing ensured the app was easy to use and that the intended clinical messages were communicated effectively.

## Introduction

### Background

The focus of this paper is on the application of methods for developing usable, effective, and engaging interactive digital health interventions (DHIs), taking as a case study an intervention, Empowering People through Informed Choices for HIV (EPIC-HIV1), intended to enable men to make an informed choice about testing for HIV and, if necessary, engaging with care. Recent literature shows that men are left behind in the response to HIV [1] as evidenced by the low uptake of HIV testing, prevention and treatment compared with women [2-4].

### Challenges of Developing Interactive DHIs

As digital technologies become more affordable, their potential is increasingly leveraged for DHIs in a broad range of settings including both affluent and resource-constrained communities, in urban, rural and remote settings [5]. There are numerous challenges with implementing DHIs such as ensuring interventions keep pace with rapidly changing technical systems [6], supporting usability needs [7], ensuring that the intervention is accessible across devices and operating systems [8], and making it culturally appropriate [9]. These challenges are exacerbated in resource-constrained settings where there are variable infrastructural constraints and levels of familiarity with technology within intervention cohorts.

There are few accounts of how to develop usable, engaging, and effective DHIs. One of the most widely cited is the person-based approach (PBA) to intervention development [10]. Although this approach describes the need to iteratively test prototypes with end users, it omits detailed discussion of interaction design and how to integrate this into the intervention development process. Conversely, the human computer interaction (HCI) literature rarely addresses issues that matter to population health researchers such as content design, clinical effectiveness, scalability, and sustainability [11]. The aim of the work reported here was to reflect on the integration of PBA and HCI methods taking as a case study the design and deployment of EPIC-HIV1, a tablet-based app.

Apps are particularly suited for stigmatized health conditions like HIV because they provide users with privacy and anonymity [12,13]. In addition, they offer health interventionists the ability to deliver uniform messages, free from embarrassment which affects people’s ability to communicate consistent and accurate information [13]. Several studies have used digital technology to increase uptake of HIV testing and medication adherence [14,15]. These studies found that both mobile and tablet-based HIV interventions are feasible, acceptable, and effective methods to engage hard to reach populations [16]. However, the studies do not discuss how the interventions were designed or developed.

### Interdisciplinary Approaches to Developing Digital Health Interventions

The World Health Organization advocates the application of user-centered design to develop DHIs to ensure that they are effective, accessible, acceptable, and user-friendly [17]. This often requires the collaboration of professionals from health and HCI backgrounds. Health care professionals, including epidemiologists and social scientists, contribute clinical expertise, theoretical understandings of behavior change and health outcome evaluation techniques. HCI professionals contribute user-centered design methodologies, which are applied to understand user needs, inform the design of digital technologies and evaluate them in terms of usability and user experience [11,18,19].

Despite the clear benefits of interdisciplinary collaboration, there are well-recognized challenges [11,18] relating to the academic heritage of the respective disciplines. Professionals from health care backgrounds put greater emphasis on measuring the effectiveness of DHIs and evaluating outcomes. HCI professionals focus on iterative design, including alternative design representations and formative evaluation, to ensure digital technology is usable and acceptable. As a result, interdisciplinary teams experience divergent approaches to timelines, measuring effectiveness and understanding success.

Literature in the field of behavior change tends to limit the integration of HCI methods, and therefore collaboration, to discrete stages in the intervention development process: for example, evaluating usability and engagement once a prototype has been developed [20].

In HCI literature, there is more interest in exploring the boundaries of the role HCI should play in DHI design and evaluation, rather than identifying discrete stages in the development process in which HCI approaches can be applied. Klasnja et al [21] describe the contribution of HCI as being to understand “how and why” DHIs work, rather than measuring outcomes. In contrast, Smith et al [22] advocate using a value chain analysis to evaluate how short-term (proximal) metrics relate to long term (distal) behavior change and health outcomes.

Marcu et al [23] describe the Patient-Clinician-Designer framework, which seeks to integrate multiple perspectives throughout the design process. In understanding the clinical context, patient needs and technical constraints, they ensure that digital interventions meet the needs of all stakeholders and are therefore more likely to be successful when implemented [23]. They describe this in reference to a mental health application, but it has also been applied to the development of applications for stigmatized conditions including HIV [24]. Similarly, Blandford [11] highlights the value of applying HCI methodologies throughout intervention development to facilitate more systematic trade-offs between what is needed for users and clinicians and what is feasible in the context.

### Person-Based Approach and Integration With Human Computer Interaction Methods

We used the PBA to inform the design and development of EPIC-HIV1. The PBA [10,20] provides guidelines on how to use qualitative research methods to identify psychosocial factors that influence the effectiveness and acceptability of interventions. Using rich, qualitative research at every stage of the intervention development process facilitates an investigation of the beliefs, attitudes, needs and situations of participants in the intervention. Yardley et al [10] propose that using PBA can make an intervention more relevant, persuasive, accessible and engaging.

The PBA consists of 4 stages, namely planning, design, development, and intervention [20]. While Yardley et al [20] recognize the value of gathering user input, the focus is on how that input informs the development of content and intervention goals. They do not describe the HCI, or user-centered design, methods that should be applied or how to translate the intervention goals and content into a usable and engaging digital product.

**Figure 1:**
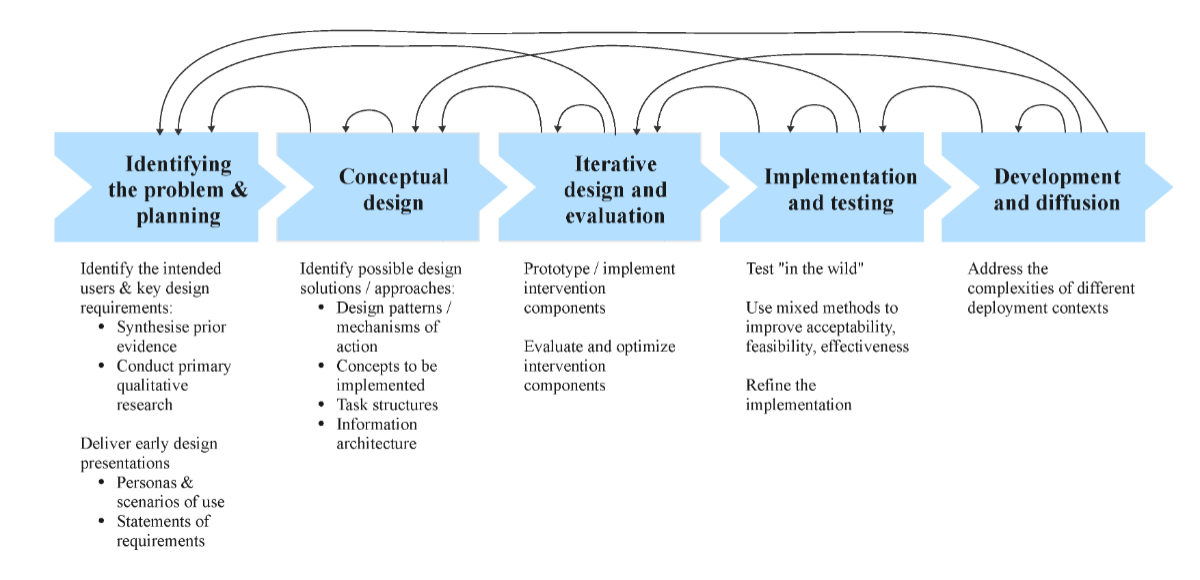
An integrated development lifecycle illustrating how a person-based approach to digital health intervention design can be augmented by human computer interaction methods.

Blandford [25] proposes an integration of PBA with established HCI design methods to address these issues (Figure 1). The key extensions to PBA are earlier consideration of design possibilities, the identification of design representations (eg, design patterns and task structures) and iteration between as well as within stages.

Both approaches advocate the use of personas and scenarios. These are rich descriptions of the intended users of the intervention and of how they will use the intervention. Scenarios are commonly presented at different levels of abstraction; for example, Rosson and Carroll [26] describe problem scenarios (the broad situation), information scenarios (giving details of the information that will be provided to users) and interaction scenarios (describing the details of user interaction with the system).

The aim of this study is to report and reflect on the process and outcomes of applying the PBA and selected HCI methods to the development of EPIC-HIV1 as an exemplar DHI for behavior change.

## Methods

### Overview

**Figure 2:**
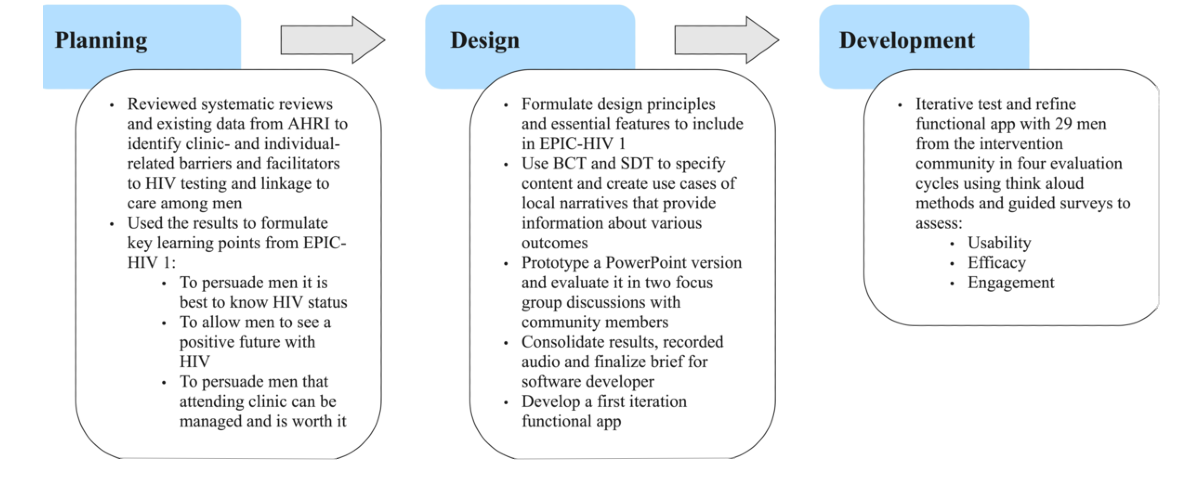
Methods used in the first 3 phases of the person-based approach, phase 4 (implementation) is out of scope for this paper. AHRI: Africa Health Research Institute; BCT: behavior change taxonomy; EPIC-HIV1: Empowering People through Informed Choices for HIV; SDT: self-determination theory.

This paper draws on documentation that was created during the iterative development process, including the initial prototyping of EPIC-HIV1 and the subsequent cycles of refinement and testing. That documentation was systematically gathered throughout the development process to support subsequent analysis. The retrospective analysis of the documentation involved the construction of a narrative of the processes and outcomes for the planning, design, and developing stages outlined in Figure 2 and presented below (after an overview of the context and rationale). Therefore, this narrative is presented as “results” below, rather than “method”. The implementation stage (ie, deployment in a clinical trial) is beyond the scope of this paper and is described elsewhere [4,27,28]. We present the context, rationale, and design aims for EPIC-HIV1.

### Empowering People Through Informed Choices for HIV Context and Rationale

EPIC-HIV1 is 1 of 2 DHIs that make up the EPIC-HIV intervention. The second DHI, EPIC-HIV2, was designed to support engagement in care for those who did not link to care within a month after a positive HIV diagnosis and is described elsewhere [29]. EPIC-HIV is 1 of 2 interventions in the Home-based Intervention to Test and Start (HITS) trial that sought to increase HIV testing and linkage to care among men in rural South Africa using micro incentives and male-targeted decision support [4,30]. The HITS trial aimed to compare the impact of micro incentives (financial) with decision support (EPIC-HIV). It was implemented in a rural setting in uMkhanyakude district in northern KwaZulu-Natal, South Africa using the Africa Health Research Institute (AHRI) Demographic and HIV surveillance platform [31]. The trial results are reported elsewhere [4,27,28].

For this case study, we focus on the development of EPIC-HIV1. As noted above, it is an educational app that introduces men to accurate information about HIV and encourages them to make an informed choice about engaging with HIV testing and care. The design drew on self-determination theory (SDT) [32] to plan content by supporting three basic psychological needs (autonomy, competence and relatedness) and increase men’s internal motivation to test for HIV and link to care where necessary. We also drew on the behavior change taxonomy (BCTv1) proposed by Michie et al [33]. They list 93 techniques that can be applied in behavior change interventions, clustered into 16 groups (eg, goals and planning, feedback and monitoring, and social support, providing a credible source of information and advice). Many of these are potentially relevant to the design of digital interventions: for example, goal setting, providing instructions on how to perform a behavior, or providing a credible source.

EPIC-HIV1 was designed to be delivered by fieldworkers during the annual household visits which includes conducting household and individual behavior and health related surveys and collecting dry blood spots for anonymized HIV testing as well as the offer of a rapid HIV test [31]. The concept was that the app would be deployed on the fieldworker tablet and offered to men during the data collection for individual surveys where the fieldworker is alone with the participant. The app is offered before the HIV rapid test offer. The fieldwork visits are carried out in a systematic cycle where they are allocated a number of households to cover in a week. Due to this time constraint, user interaction with EPIC-HIV1 was allocated 10 minutes.

### Design Aims for Empowering People Through Informed Choices for HIV

Two key aims for EPIC-HIV1 were ensuring that it met users’ needs, that is, that participants were engaged by the content and could autonomously navigate the application, including people with low digital and health literacy; and ensuring that it met the intervention goals, namely to communicate the correct clinical messages to men and enable them to make an informed choice about engaging with HIV care.

The design had to recognize the stigma related to HIV [15,16,34]. This relates to both the design of the intervention itself, and the evaluation methods used. For example, in user studies we did not require participants to disclose their HIV status. Also, to enable study participants and future users to explore EPIC-HIV1 independently and privately, earphones were offered for their use.

Another design challenge in resource-constrained settings is low literacy [35]. Solutions proposed for low-literacy users include graphical interfaces, interactive voice interfaces, and mediated usage [36]. Designing for low literacy populations extends beyond catering for users not being able to read, to multiple cognitive and emotional factors that influence how users engage with content [37,38]. For health care interventions, there is added complexity: individuals with lower literacy tend to have lower health literacy [39], and having lower health literacy puts an individual at higher clinical risk and is associated with reduced ability to exert control over health through informed decision making [39,40]. The approaches taken in both design and testing to be inclusive of users with low digital and health literacy are discussed below.

### Ethical Considerations

This study received ethical approval from the University of KwaZulu-Natal Biomedical Research Ethics Committee (BFC398/16). Participants involved in empirical data collection provided written or verbally informed consent before participation. Participation in the study was entirely voluntary. To protect participants’ privacy and confidentiality all data were deidentified, and no personally identifiable information was included in the final dataset. Participants received light refreshments (sandwich and juice) for taking part in the study and no financial rewards were provided.

## Results

### Overview

As noted above, EPIC-HIV1 was developed following the first 3 stages of PBA. The planning and design stages were led by social scientists and health professionals (JS, SW, MS, TZ, PM, and TM) and the development phase was led by HCI specialists (AB and AZ) and the technology partner. Each stage used different recruitment and sampling methods as described in detail below.

### Person-Based Approach Stage 1: Planning

The planning stage is described in detail by Mathenjwa et al [29]. In summary, we reviewed systematic reviews and existing data from AHRI to identify barriers to HIV testing and linkage to care and used the results to define the user requirements of the app. In summary, key barriers to testing were fear of rejection, and being blamed for sexual misconduct, if found to be HIV positive; fear of death or illness; feeling healthy and so not understanding the need to test; a preference for traditional medicine; and a belief that medicine should be curative (rather than for preventing or managing illness). Key barriers to engaging with care were that clinics were regarded as feminized spaces where men do not feel comfortable; that they are concerned about confidentiality if they are seen in clinics; that there may be long wait times in clinics; and that they often have poor relationships with health care workers. Key learning points for EPIC-HIV1 were that it was important to persuade men that it is best to know their HIV status, because it allows them to take control; that men needed to see a positive future with HIV, in which they are in control and can achieve their ambitions, and that attending clinic can be managed and is valuable in the long term.

### Person-Based Approach Stage 2: Design

The aim of this stage was to formulate the design principles of the application and essential features that should be included. Using the results from the planning stage, we identified barriers that can be modified by EPIC-HIV1 and key learning points to be incorporated into the app. We then formulated intervention features relevant to the learning points. Finally, we identified specific behavior techniques from the BCT taxonomy [33] to support autonomy, competence and relatedness. The components are interrelated as illustrated in Table 1.

**Table 1:** Key learning points from the planning stage of person-based approach and how they relate to self-determination theory and behavior change taxonomy.

Learning point	SDT^a^ relevant	BCT^b^ from taxonomy
To persuade men it is best to know your HIV status, because it allows you to take control	Persuading users that knowing their status allows them to take control of important aspects of their lives, such as upholding traditional values, caring for a family or having sex and children (supporting autonomy).	9.1 credible source 5.1 information about health consequences 5.2 salience of consequences
To allow or engage men to see a positive future with HIV, in which they are in control and can achieve their ambitions	Maintaining a positive illness context throughout: that it is possible to live with HIV and do all one wants to do. Educating men about HIV and the benefits of testing and taking antiretroviral therapy (supporting autonomy).	9.1 credible source 5.1 information about health consequences 5.2 salience of consequences 13.5 identity associated with changed behavior 13.1 identification of self as role model
To persuade men that attending the clinic can be managed and is worth it in the long term	Persuade men that they can handle going to the clinic through positive examples of men talking about what it was like and how they managed it (supporting relatedness, competency and autonomy).	13.5 identity associated with changed behavior 13.1 identification of self as role model

^a^SDT: self-determination theory.

^b^BCT: behavior change taxonomy.

#### The Conceptual Design and Scenarios of Use

The high-level design concept was to provide experiential information from local men living with HIV that users could identify with. The content was rooted in local narratives to provide information about various outcomes to increase risk perception, salience, and likelihood of response by making the decision to test or link to care explicit. Personas were implicitly encapsulated in the descriptions of the characters who were to be introduced through EPIC-HIV1: men of different ages and with different health beliefs and levels of digital literacy.

As described above, the envisaged context of use for EPIC-HIV1 was that it would be administered by the AHRI fieldworker during the annual individual biobehavioral surveillance home visit. When the time came to explore EPIC-HIV1, the fieldworker was expected to hand over the tablet computer with the app opened (start screen) to the user (consenting man), together with earphones (that the man could keep) and wait while they interacted with the app in private. The app was designed to take 5-10 minutes depending on the pathway that the user selected to align with the time constraints of the fieldwork.

The information scenario was based on the notion that the app should acknowledge and refute common misconceptions about HIV (how it is transmitted and what treatments are effective) and concerns about attending a clinic and making other people aware of their HIV status.

The interaction scenario involved the user working through 3 sections (illustrated in Multimedia Appendix 1. First, there was a short introduction, after which the user was given the choice to either find out about HIV testing and treatment (if they were not ready to test) or just find out about treatment (if they were ready to test). In the testing section, users were given the option to listen to short vignettes describing common reasons for men not wanting to test for HIV. Users were then presented with information, addressing each of these reasons and providing a counter argument. In the treatment section, 3 characters described their journey since being diagnosed with HIV; these characters represented different personas, and the user could listen to one of their stories (ideally the one they found most relatable).

All app content and instructions were provided in text and audio format. Still images, including photographs of individuals (with faces obscured) and artefacts from the intervention community, supported the audio descriptions.

**Figure 3:**
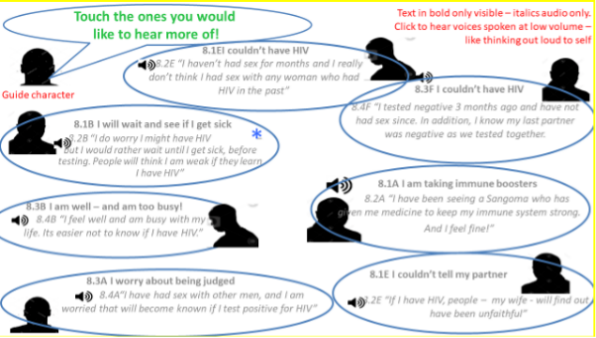
Illustration of 7 characters from the PowerPoint prototype of Empowering People through Informed Choices for HIV.

The app used a male nurse from AHRI to serve as a guide character and credible source of HIV information. He gives an introduction and explains how the app works and then reads out the 2 options (“I am sure I will have an HIV test today” and “I might not have an HIV test today”) and asks the user to select which statement he agrees with. In the “ready to test” section, the nurse applauds the user for taking the decision to test today. In the “not ready to test” section, we used 7 characters to give short vignettes discussing common reasons for men not wanting to test for HIV which were identified during the planning stage. The nurse comes back to address the reason each character lists, providing a counter argument. Users can then go into more detail by selecting one of the 3 main characters that persuade men that knowing their HIV status gives them control of important aspects of life, that it is possible to live with HIV and still achieve goals and finally that they can manage going to the clinic (Figure 3).

#### Testing the Conceptual Design

We developed a low fidelity PowerPoint (Microsoft Corp) protype of the app and evaluated it with 2 focus groups of men from the community advisory board, a body representing members of the intervention community that acts as a bridge between AHRI and the community, safeguarding the rights of the study participants. We sought to include younger and older community advisory board members. The focus of the evaluation was on whether the characters and messages selected were acceptable to men in our community. Since we aimed to assess acceptability of the content and characters, findings were categorized into content or character related and the app functionality (Table 2).

**Table 2:** Summary of the focus group discussion results and how we addressed them.

Main issues	Issues	Steps taken and revision of content
Content	Self-stigmatization after testing HIV-positive. Fear of weakness because of illness. Manhood is about becoming isoka (Casanova). Testing is for everyone, not for particular groups or types. Most men want to wait until they get sick before testing, long queues in clinics, feminized spaces of health care.	Positive reinforcement on autonomy, competence and relatedness among all characters.
Characters	Groups preferred characters to look like strong men. Groups commented on what characters should wear. Groups mentioned that characters’ gestures should be open and clear.	Only the nurse character will be seen by users. No other faces will be shown, real settings will not be used. Men wearing what was proposed in groups. Characters’ gestures open and clear.
Functionality	Asked for a rewind button so that people can go back and listen. Privacy and confidentiality.	No rewind option because of time. App will be private, and fieldworker will clarify to each participant that they will not know how the participant navigated the app.

**Figure 4:**
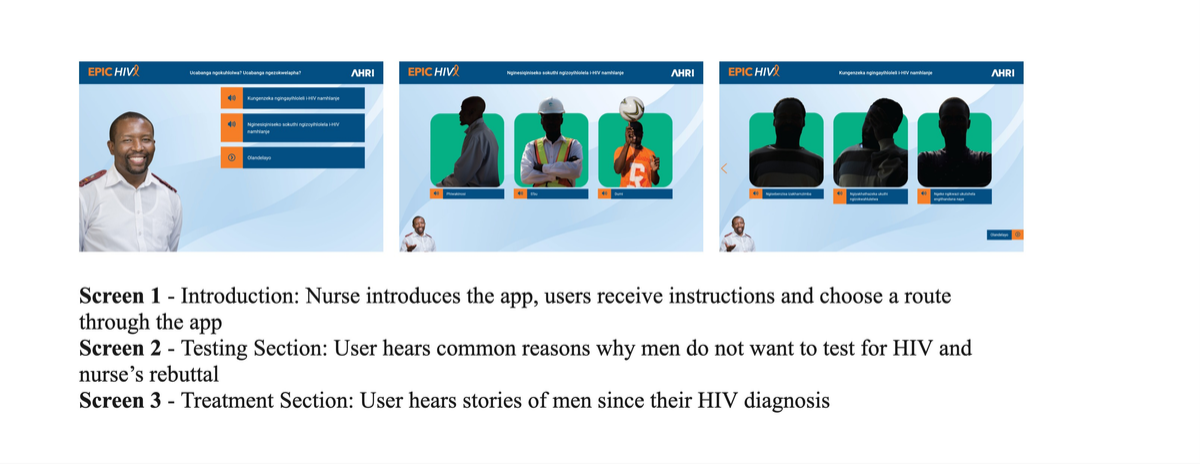
First iteration of the Empowering People through Informed Choices for HIV app cycle (3 example screen shots).

We used the results to refine the content and characters and finalized the brief for the software developer. We selected a cast of actors from the local community to represent the different characters and did an audio recording of voice overs and a photo shoot at AHRI offices and a local community center. This was sent to the software developer with the brief. We received the first iteration of a functional app, as illustrated in Figure 4; however, it was not considered by the team to be engaging.

### Person-Based Approach Stage 3: Iterative Testing and Design Changes

We iteratively tested and refined EPIC-HIV1 to ensure that the app was interactive, usable and acceptable for a broad spectrum of users with varying education and digital literacy. In total, 4 cycles of user-centered evaluation and design were conducted, based on design sprints each lasting 2 weeks. These cycles were structured using a simple interaction design model [41], whereby initial user requirements were identified, design improvements were generated, and the new design was evaluated. The evaluation metrics are summarized in Table 3.

**Table 3:** Summary of evaluation metrics relating to usability, efficacy, and engagement.

Metric	Questions	Method
Usability	Is it clear how to move between screens? Is it clear how to engage with the information on each screen (ie, choose options, listen to audio and exit audio)?	Observations during think-aloud sessions
Efficacy	Are they key messages communicated? Do participants gain new information because of using the app? Is the information they gain factually accurate?	Questions in think aloud protocol and guided survey
Engagement	Is the interaction pleasant? Is the presentation of content appropriate and interesting?	Questions in think aloud protocol and guided survey

During each cycle, participants were asked to complete defined tasks and articulate their thoughts about EPIC-HIV1 and the task. Both actions and verbalizations were recorded to identify the main usability challenges and build an understanding of the acceptability of content. Participants were paused after the introductory screens and asked questions regarding their initial expectations of the app’s content and purpose. After using the app, participants were asked further questions of their understanding of the content and reflections on the design. This guided, verbally administered, survey ensured that we did not exclude low-literacy participants. It consisted of a mix of open and closed questions and was used to gather insight into usability, comprehension, and design preferences. The evaluations were conducted in isiZulu, with a bilingual (isiZulu-English) researcher administering procedures and participants were observed as they used the application.

#### Recruitment and Participants

All participants were recruited from the AHRI demographic and HIV surveillance community. Participants were approached in public spaces by a local male researcher from the community. These public spaces varied but included parks, taverns, and the roadside. Across the 4 evaluation cycles, sampling became increasingly purposive, focused on more rural areas and older participants, as we sought to recruit participants who represented specific user groups within the intervention cohort, especially those who had been identified as likely to have low technological literacy.

We conducted evaluations with 29 unique isiZulu speaking males (excluding pilot participants) over the 4 iterative cycles. All participants were asked their age to confirm they were aged 18 years or older and able to consent to participate. Recruitment was helped significantly by the presence of local researchers who knew where men in the community “hang out” and were able to explain the purpose of the study. Men were often in groups and many declined to engage, meaning all 29 participants engaged willingly.

#### Overview of Evaluation Cycles

Across the 4 cycles, the evaluations became more refined, but the objectives and the metrics were consistent. Correspondingly, there was consistency across the protocols. For example, some of the questions (Table 3) were the same and were asked at the same point in the evaluation. The key features of each evaluation cycle are summarized in Table 4 and refer to Multimedia Appendix 2 for example of findings and changes made in each evaluation cycle.

**Table 4:** Key features of each evaluation cycle.

Evaluation cycle	Objectives	Methods
Evaluation 1	Primary: identify main usability challenges, gather understanding of target user group’s needs, and assess understanding of content. Secondary: gather insight into potential design improvements.	Think aloud. Observation. Guided survey.
Evaluation 2	Primary: make layout easy to use for people who are novice tablet users and have low literacy, assess understanding of content. Secondary: gather insight into preferred look and feel of app.	Observation. Retrospective usability questions. Guided survey.
Evaluation 3	Primary: test whether users know how to select options, assess understanding of content, make content more engaging. Secondary: ensure that uninterrupted participants were completing the app in an appropriate time.	Observation. Retrospective usability questions. Guided survey. Task completion times.
Evaluation 4	Primary: assess whether the changes made to the application affected users’ expectations, such that they were making more informed decisions.	Observation. Retrospective usability questions. Guided survey.

#### Specifics of Each Evaluation Cycle

For the first evaluation, convenience sampling was used to recruit from the community, working in or close to the research institute. The evaluation took place within the research institute. After interacting with the first version of the app, participants were presented with low fidelity interactive prototypes of different sections of the app and asked which they preferred, which was most relatable, and which was most clear.

**Figure 5:**
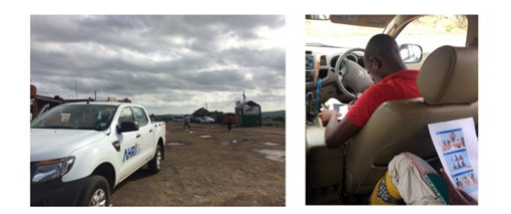
Illustrative photos of pop-up evaluation testing in remote locations using an Africa Health Research Institute vehicle.

In Evaluation 2, we used pop-up research [42], which involves carrying out targeted user evaluations in environments that are familiar and used by the app’s intended audience, to simulate some of the environmental constraints and social considerations that would be experienced by fieldworkers when administering the DHI, such as lack of access to the internet and difficulty finding private spaces. This had the potential to reduce participant response biases [37] by making the environment more familiar and less formal than the research institute. Participants were approached in public spaces, such as at bus stops or tuck shops, and evaluations were conducted either in those spaces or close by in the researchers’ car (illustrated in Figure 5). These decisions helped to establish a level of mutual trust and collaboration between the researchers and participants. Unlike most accounts of pop-up research, the planned time for each individual’s participation was not particularly short: it lasted 20-30 minutes, sometimes more if the participant had low literacy.

Based on learnings from Evaluation 1, we administered retrospective usability questions rather than asking participants to articulate their thoughts concurrently. We also printed screenshots of design variations on paper and described the differences to participants then asked them which they preferred, rather than continuing to use low-fidelity interactive prototypes after the first part of each session. We included closed questions that were asked while participants were interacting with EPIC-HIV1: for example, we added a help button; in the evaluation, we stopped users after the instruction screen and asked them to indicate which button to press if it was not clear what to do next. Most participants were unaware they could skip content or be selective, which indicated that the app was not supporting autonomous decision making. When presented with design alternatives, all 6 participants selected the interface with photo images because it was “clear.” This supported the use of rich and contextually relevant images throughout the app to optimize the relatability of the content.

**Figure 6:**
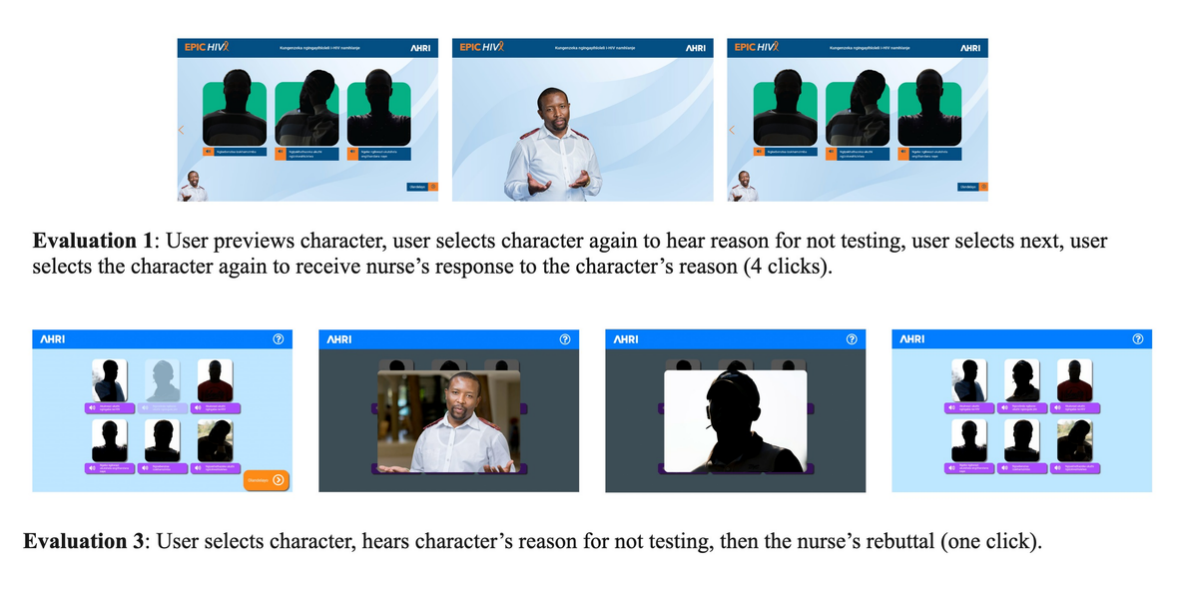
Example of design changes made, illustrated with screenshots from the first and third versions of Empowering People through Informed Choices for HIV.

Following Evaluation 2, previews were either automated or removed, which meant users would only be presented with choices when it was critical. We added extra instructions on how to select options, made button states more distinct and provided additional nudges in the form of button animations and instructions from the nurse character. Additional photographs of paid professionals who were representative of the intended cohort were added to the character stories to make them more engaging (Figure 6).

In Evaluation 3, as well as assessing whether participants knew how to select options, understood correct messages and found the content engaging, we wanted to ensure that participants were completing the app within a given time. We split participants into 2 groups: one was interrupted to assess their option selection while the other was uninterrupted, to gather their task completion time and observe the full user flow. Having identified rural and older people as those who were least comfortable using the app, we actively recruited males representing this category, reducing the proportion of young periurban males (the easiest to find and recruit). After conducting evaluations with 12 participants from various locations, we had reached saturation in responses. Compared with earlier evaluation cycles, we focused more on option selection and expectations. For example, at the beginning participants were asked what they had expected to find if they had selected the other option so that we could assess whether participants were actively choosing what they wanted to hear about. This was particularly important because users could only engage with EPIC-HIV1 once as part of the planned intervention and could not return to content of interest.

Evaluation 4 did not reveal any additional usability issues. Participants were able to describe what they would have had if they had selected the other option, indicating the changes to the language used at the beginning of the app were effective and supported informed decision making.

### Summary

Both the evaluation objectives and the methods became more refined during the process. For example, the objective of the first evaluation was to get a sense of what users were finding difficult and what evaluation style would be contextually appropriate. Convenience sampling was used to recruit participants and the questions in the evaluation were relatively general. In the third evaluation, 2 of the objectives were to assess whether participants could make accurate option selections and complete the app within a given time. Purposive sampling was used to recruit participants who represented a range of users we had previously encountered, especially those who had had usability challenges (ie, low literacy, novice users, and older people). The evaluation was more focused, questions were more specific, and the methods were altered to support our objectives.

Although pop-up research was an effective and valuable means of recruiting participants and conducting evaluations, we had to ensure that we adequately addressed considerations, including ethics (ensuring the participants did not feel coerced), privacy (ensuring that participants could freely engage with content without passers-by hearing), community-institution relationships (avoiding contributing to “participant fatigue” near the research center), and our own safety as we went into remote places and invited people into our car or worked with them in unfamiliar environments.

## Discussion

### Principal Findings

The analysis of existing theory and data enabled the team to identify and address the most important barriers to men engaging in HIV testing and care in a cost-effective way. The use of HCI methodologies enabled the team to translate the intervention goals, that is, communicating persuasive messages in a way that would promote behavior change, into design strategies and ensure the application was usable. The resulting application was inclusive of the intended population, supported meaningful choice in how users engaged with the application, and ensured the correct clinical messages were communicated.

One key lesson from this study is that it is essential to design the introduction (or “onboarding”) to a DHI very carefully, to ensure that users are empowered to use the app effectively and that expectations of what they will experience are set appropriately. Another key lesson is that an appropriate balance had to be achieved between autonomy (enabling people to navigate as they choose) and ensuring that the correct clinical messages are communicated.

The conceptual design featured a series of dialogues between relatable characters and a trustworthy nurse. These dialogues were accompanied by images of the characters in familiar settings. However, this approach was never tested against alternative design strategies. Early decisions about the design and mode of the interaction were based on informal understanding and intuition rather than evidence. Resource limitations (both time and funds) meant that we did not have the capacity to revisit earlier design decisions such as the appropriateness of using a tablet computer or the general approach of presenting dialogues. Thus, it was not possible to engage in the broader cycles of iteration proposed by Blandford [25].

Early design decisions, such as the approach of focusing on a dialogue between relatable characters and a trustworthy nurse, and about the content of the dialogue, were based on the key scenario of use: that is, that the tablet computer would be lent to the individual for up to 10 minutes within the annual health visit; this imposed a significant constraint on the design. For example, the main interaction design issue raised by members of the community advisory board in the Stage 2 testing, that people would like a “rewind” option, was not addressed due to this time constraint.

Through iterative testing the main area of concern, and where many changes were made, were in the introduction, or “onboarding,” for the app. It was found necessary to set expectations about the app (eg, that it would not deliver an HIV test result) and to provide detailed verbal instructions about how to select options and press buttons on a touch screen.

We needed to balance the needs for the app to be engaging and inclusive. The participants were diverse, and levels of technical, educational and health literacy varied widely. Some participants were comfortable using the tablet app and owned touch screen smartphones. Testing highlighted the tension between providing literal choice in interaction design and meaningful choice regarding how the user engaged with the intervention. For example, giving users a choice about whether to listen to the nurse’s response after hearing a character’s concerns proved counterproductive as it did not support the concept of “informed choice,” so the choice point between concern and response was removed after the first evaluation cycle.

Changes to the design were intended to support low-literacy users: for example, by adding audio instructions, highlighting one option at a time, and reducing the amount of user input required. However, we maintained critical choice and ensured it was well understood. The reduction in user input was balanced with ensuring that the app remained engaging and persuasive for participants with higher literacy levels. Through iterative cycles, we were able to deliver a design that was inclusive of as much of the target cohort as possible.

Nevertheless, we had to recognize that the delivery of the intervention was likely to exclude a small proportion of the potential beneficiaries. For these people, for whom the app was inaccessible, a conversation with the visiting health worker would be a possible alternative.

### Limitations

As noted above, HCI methodologies had not been involved during the early stages of intervention development. As a result, the remit of HCI was to fix the app, to make it as good as possible given the time and resources remaining. We believe the app design could have been stronger if HCI methodologies had been used alongside PBA and BCT approaches from the outset. For example, there was no opportunity for exploratory research to define what interactivity meant for the intended population or understand whether a tablet-based app was an appropriate approach to communicating HIV/AIDS information in this context, or whether it could inadvertently bias participant responses, as indicated by previous research [43], or exclude a proportion of the intended target population.

There was also no opportunity for HCI focused summative research, for example, to understand how users had navigated through the app, areas of interest or potential drop-off points [21]. The HITS clinical trial results [4,27,28] suggest that EPIC-HIV1 did not increase men’s uptake of HIV testing. This raises many questions that could be the focus of future research: for example, would a different kind of digital intervention have been more effective, was the approach of fitting the user’s interaction with the intervention into the annual visit effective, are digital interventions inappropriate in this context, or does the intervention need to be better tailored toward different subpopulations (eg, those with different levels of digital literacy or different priorities in life)?

### Conclusion

The aim in developing EPIC-HIV1 was to support a clinical trial investigating whether financial incentives or a digital intervention was more effective than the established approach to encouraging men to test for and engage in care for HIV. The implicit assumption was that any digital intervention would be equally effective and no comparison between different digital interventions was planned.

By applying an iterative design approach, we were able to make systematic choices regarding design that would facilitate usability, engagement, and comprehension required for the intervention. The value and necessity of these choices were brought into sharp focus with this case study as many of the challenges of developing an DHI were exacerbated by the study context (resource constrained, low digital literacy, and stigmatized condition).

Developing Digital Health as an interdisciplinary field requires close collaboration throughout the intervention design process to ensure that the resources and expertise required are available in a timely way.

## Appendix

Multimedia Appendix 1 Overview of user journey through the app.

Multimedia Appendix 2 Example of findings and changes made in each evaluation cycle.

## Abbreviations

AHRI: Africa Health Research Institute
BCT: behavior change taxonomy
DHI: digital health intervention
EPIC-HIV: Empowering People through Informed Choices for HIV
HCI: human computer interaction
HITS: Home-based Intervention to Test and Start
PBA: person-based approach
SDT: self-determination theory

## Data Availability

The datasets generated in the study are available from the HITS trial principal investigators on reasonable request.
