# Supplementary figures and images for: Creating a Usable and Effective Digital Intervention to Support Men to Test for HIV and Link to Care in A Resource-Constrained Setting: Iterative Design Based on A Person-Based Approach and Human Computer Interaction Methods
Source: JMIR Form Res. 2025 Apr 17;9:e65185. doi: 10.2196/65185 (PMC12046270; doi:10.2196/65185)

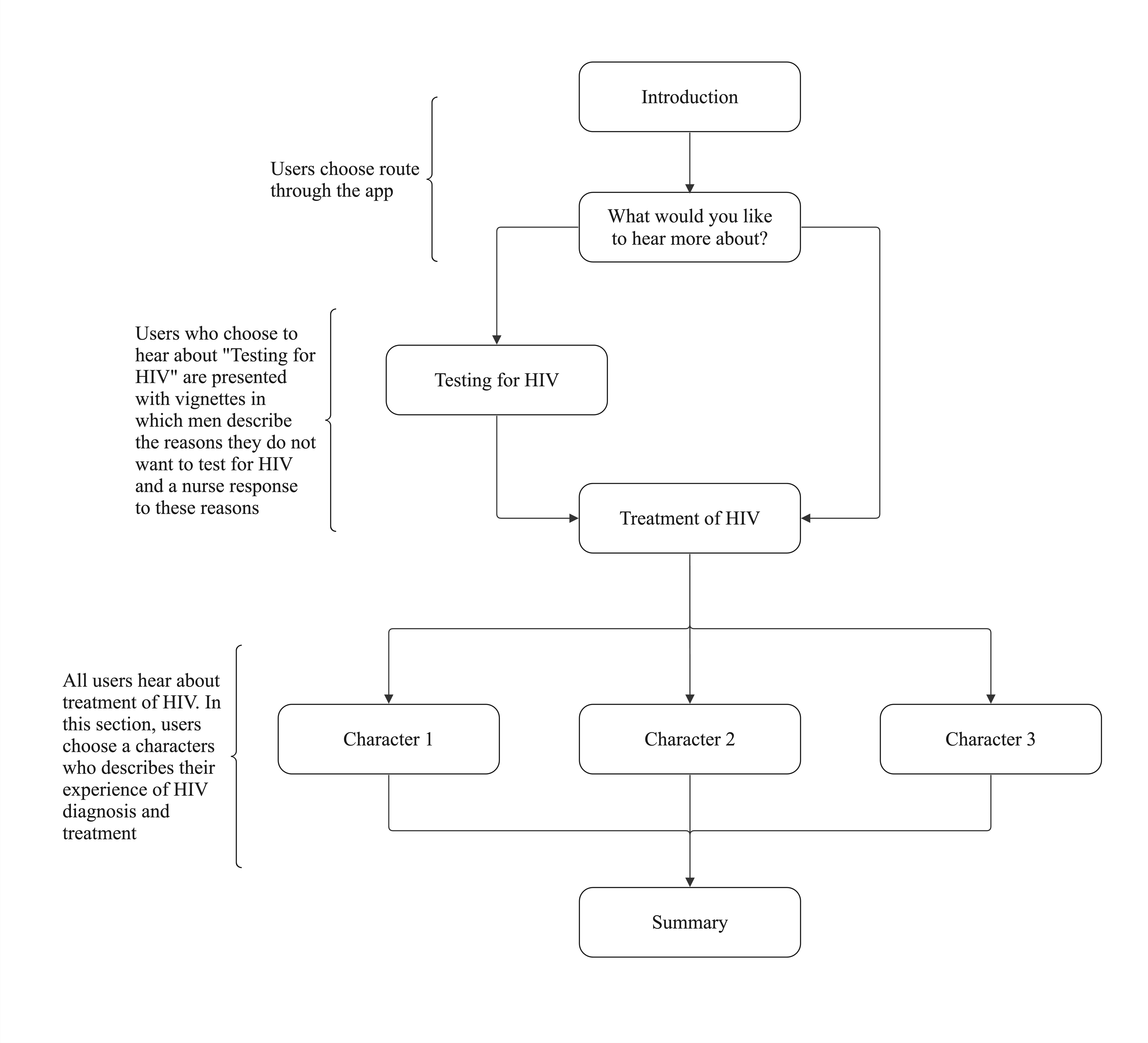

Supplement: Multimedia Appendix 1 [file formative_v9i1e65185_app1.png]
