# Supplementary material for: Creating a Usable and Effective Digital Intervention to Support Men to Test for HIV and Link to Care in A Resource-Constrained Setting: Iterative Design Based on A Person-Based Approach and Human Computer Interaction Methods
Source: JMIR Form Res. 2025 Apr 17;9:e65185. doi: 10.2196/65185 (PMC12046270; doi:10.2196/65185)
Supplement: Multimedia Appendix 2 [file formative_v9i1e65185_app2.docx]

| **Evaluation cycle** | **Example findings** | **Example changes made** |
| --- | --- | --- |
| **Evaluation 1** | The objective of the app was unclear: some users expected the app to check their HIV status.  Users don’t know how to make option selection throughout the app. | Introductory information and additional instructions added to clarify the purpose of EPIC-HIV1 and how to use it. Onboarding split across three screens. |
| **Evaluation 2** | Users not clear how to initiate use of the app.  Users did not consistently understand option selection at the start of the app.  Users not able to make informed choices about how much content they listen to or whose stories they wanted to learn more about (instead listening to every piece of content but in unintended orders).  Users gathering some incorrect messages.  Preferences for images of people in context. | A call to action was added to the first screen.  Key segments of audio stitched together. |
| **Evaluation 3** | Users did not consistently understand option selection at the start of the app.  Users lost interest during longer sections of audio.  Users making informed option selection regarding which character stories to listen to.  Users understanding correct key messages.  Users completing the app within the target time. | Descriptions of options re-worded and additional audio added at the start of the app.  Shorten length of character stories. |
| **Evaluation 4** | No significant new usability issues emerged. | None. |
